# Stable isotopes and community surveys reveal differential use of artificial and natural reefs by South Florida fishes

**DOI:** 10.1016/j.heliyon.2021.e07413

**Published:** 2021-06-26

**Authors:** Christopher A. Blanar, Joseph R. Hornbeck, David W. Kerstetter, Amy C. Hirons

**Affiliations:** a Department of Biological Sciences, Halmos College of Arts and Sciences, Nova Southeastern University, 3301 College Avenue, Fort Lauderdale, FL 33314 USA; b Department of Marine and Environmental Sciences, Halmos College of Arts and Sciences, Nova Southeastern University, 8000 North Ocean Drive, Dania Beach, FL 33004 USA

**Keywords:** Reef fish, Stable isotopes, Artificial reef, Trophic dynamics

## Abstract

Artificial reefs may enhance the biological production of reef-associated flora and fauna, but their trophic structure relative to that of natural reefs remains understudied. We assessed trophic relationships by 1) comparing reef fish communities and 2) comparing δ^13^C and δ^15^N in 43 fish species from both artificial reef sites and adjacent natural reef tracts in Broward County, Florida. We tested the effect of sampling location (artificial, first, and second reef), general feeding strategy (herbivore, omnivore, invertivore, and carnivore), phylogeny, and standard length on δ^13^C and δ^15^N. The reef fish communities of the artificial and natural reef tracts were significantly different; the artificial sites also exhibited more variability. For all samples, δ^13^C and δ^15^N ranged from -19.5 to -13.1‰ and 6.7–13.3‰, respectively. Significant effects were detected for both general feeding strategy and phylogeny. Significant differences were also seen in δ^13^C and δ^15^N profiles between artificial and natural reefs; however, these changes were primarily driven by differences in fish community structure, rather than by changes in the feeding strategy or trophic relationships of individual fish taxa. The trophic guild invertivore was the only group of fish to demonstrate significant isotopic differences between both reef tracts (inner and outer) and reef types (artificial and natural). The artificial reef may act more as a foraging corridor between the natural first and second reef tracts for omnivores and carnivores. If the function of artificial reefs is to provide additional foraging habitat for fishes, then perhaps more time is needed for the trophically important, infaunal invertebrate community to develop similarly to the natural reef environment.

## 1 Introduction

Coral reef ecosystems are being degraded by anthropogenic processes including coastal development, deleterious fishing practices, and climate change; the resulting habitat loss has been associated with decreased biological production and diversity (Stone, 1985; Paddack et al., 2009; Koeck et al., 2014; Lönnstedt et al., 2014). In response, artificial reefs have been established in an attempt to mitigate the negative ecological impacts associated with the loss of natural reef habitat (Koeck et al., 2014). In the United States, artificial reef fabrication and deployments are overseen at the federal level by the National Marine Fisheries Service (NMFS) with the intent to maximize stakeholder use and minimize negative environmental impacts (Stone, 1985). Several studies have shown that the establishment of an artificial reef has the potential to create new habitat and enhance biological production (e.g., Bohnsack and Sutherland, 1985; Sheehy and Vik, 2010; Broughton, 2012). Artificial reefs have been shown to provide additional substrate for infaunal and epibiotic prey, as well as shelter, breeding, and nursery habitats for marine animals, including fishes (Connell and Glasby, 1999; Zalmon et al., 2014).

Understanding the trophic relationships within ecological communities is key to understanding community structure, including its overall ecological health and resilience (Manteufel, 1961; Hooper et al., 2005; Carscallen et al., 2012). The traditional technique used in trophic studies is gut-content analysis, which characterizes the diet of an individual by examining the contents of the stomach or full alimentary canal (Bowen, 1996; Jennings et al., 1997). However, such studies often are complicated by the unidentifiability of gut contents (e.g., detritus) and the significant overlap in prey preference among generalist feeders (Valdés-Muñoz and Silva Lee, 1977). Consequently, alternative approaches for assessing diet have been developed, including stable isotope analysis. The isotopic ratios C^12^/C^13^ and N^14^/N^15^ are most frequently used for trophic studies of marine fauna (Layman et al., 2012). Values of δ^13^C allow inference of the major sources of carbon in a food web (France, 1995a, 1995b; de la Morinière et al., 2003; Wyatt et al., 2012) because there is little isotopic fractionation associated with δ^13^C (0.5–1.0‰) between trophic steps (DeNiro and Epstein, 1978). Conversely, δ^15^N values can be used to infer trophic patterns, as an individual is enriched relative to its food source by 3–4‰ (DeNiro and Epstein, 1981; Elsdon et al., 2010). The use of both stable isotopes (δ^13^C and δ^15^N) to study the trophic behavior of reef-associated fish is well-established (e.g., Cresson et al., 2014; Post 2002). Stable isotopes have been used to assess trophic patterns before and after artificial reef construction; for example, Zhang et al. (2020) noted changes in marine food web structure, including a general decrease in fish trophic levels, and focusing of trophic level among omnivorous and carnivorous fishes. However, few studies have used stable isotopes to compare feeding behaviors in artificial versus natural reef habitats. Such studies, which are lacking, would help assess the effectiveness of the ability of artificial reefs to functionally replicate natural ones.

Florida is ideally situated to address this knowledge gap: it has the only coral reef system located within the continental United States, as well as the largest number of permitted artificial reefs (Adams et al., 2006). The community composition of southeast Florida's sub-tropical reefs generally resembles that of Caribbean and tropical Atlantic reefs (Banks et al., 2008). The aims of this study were 1) to broadly assess frequentation of artificial and natural reefs by reef associated fishes using visual community surveys, and 2) to compare δ^15^N and δ^13^C values of reef-associated fishes in both habitats, using these data to infer the extent to which trophic interactions might be altered in artificial reefs.

## 2 Results

### 2.1 Fish community composition and habitat use

**Figure 1:**
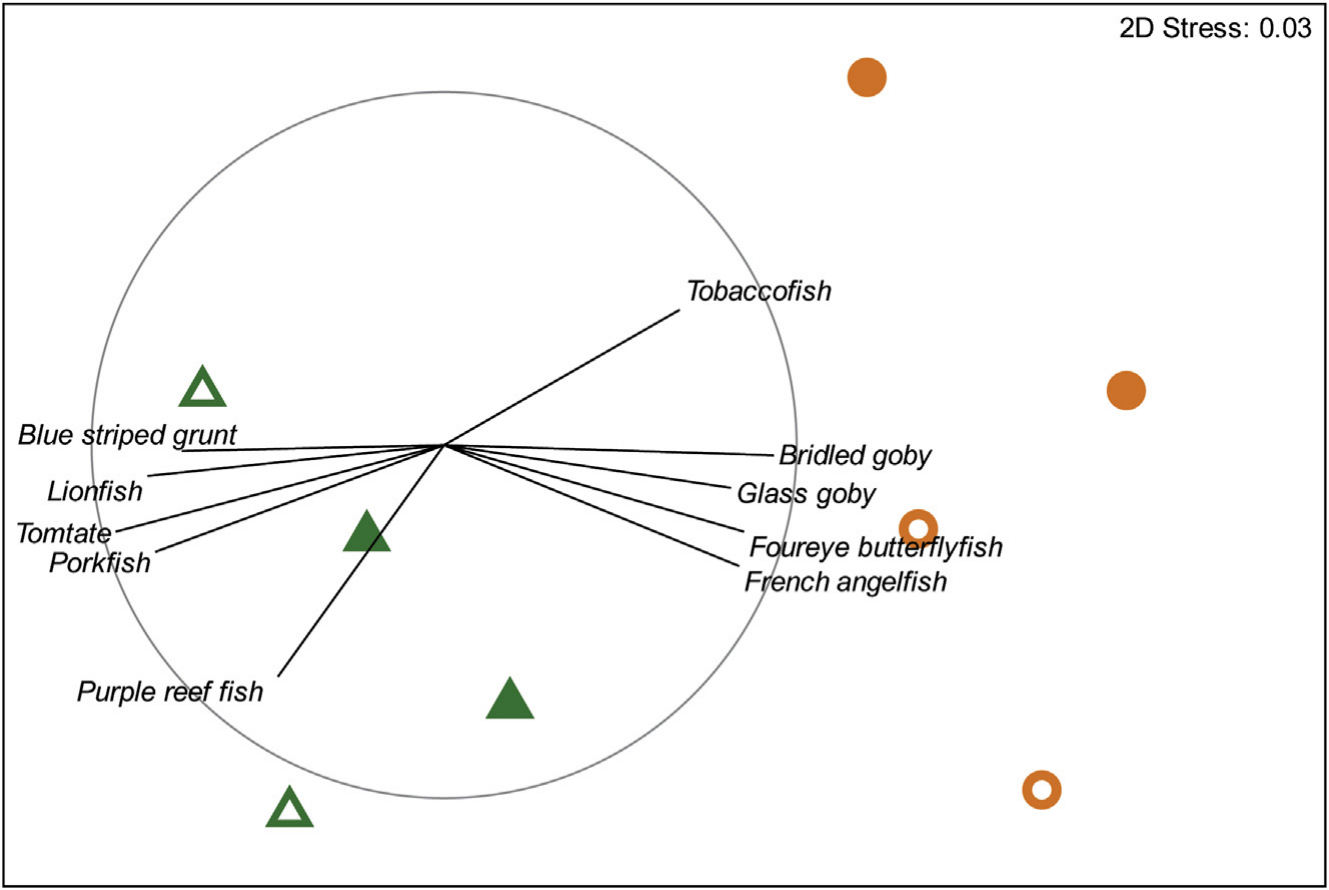
Nonmetric multidimensional scaling (MDS) plot of Bray Curtis similarities of fish communities associates with artificial (triangles) and natural (circles) reefs. Distance from shore is also indicated via outer (solid) and inner (outline) reef tracts, respectively. Pearson correlation vectors for the ten fish species most characteristic of each reef type (per SIMPER analysis) are provided as well.

**Figure 2:**
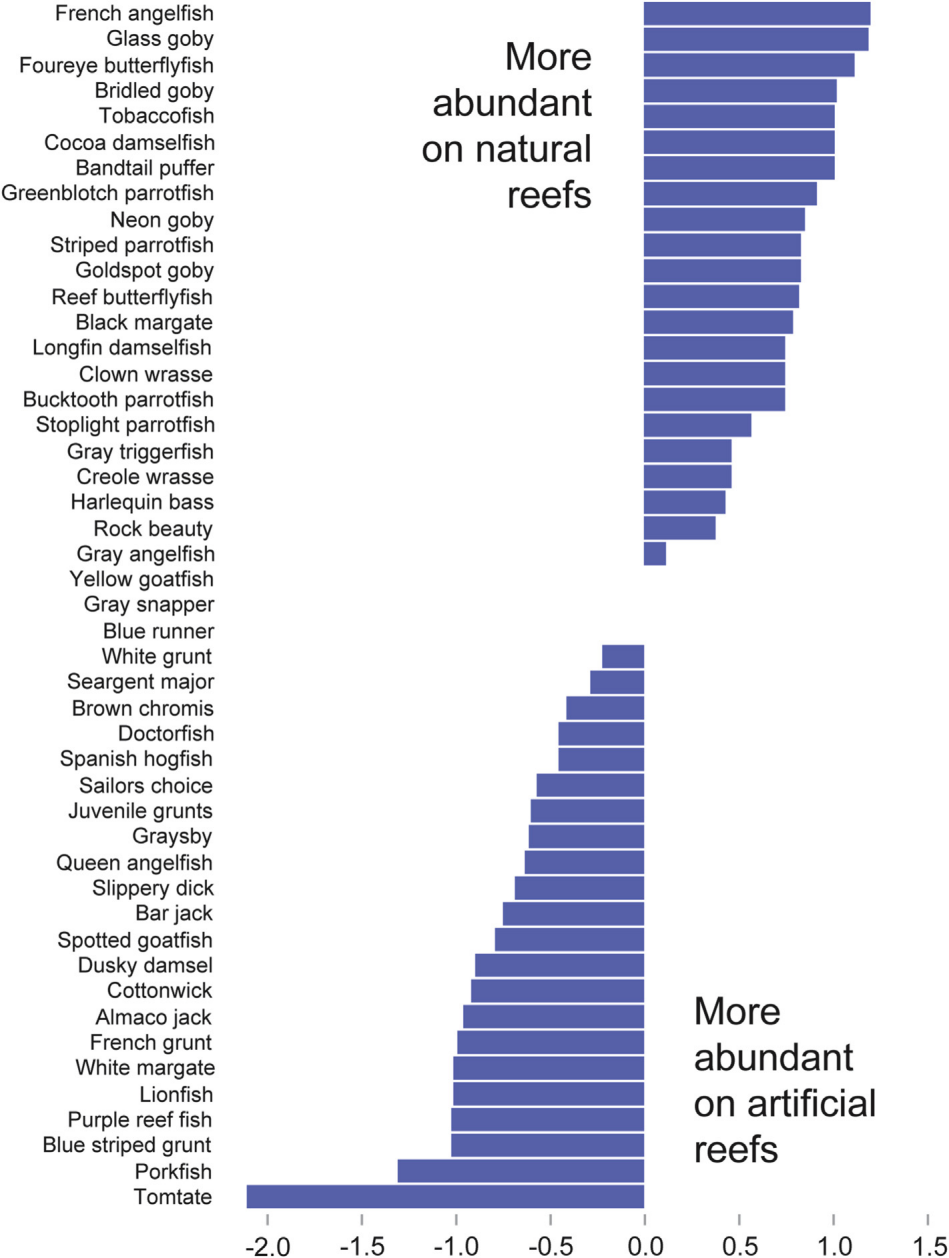
Relative abundance of fish species on artificial and natural reefs. These fish species were retained because SIMPER analysis identified them as being characteristic of each reef type. Fishes are ranked based on their preference for natural reefs. Scale on *X axis* indicates the difference in mean (square root transformed) abundance values on natural vs. artificial reefs for each species.

Analysis of visual survey data revealed that artificial and natural reefs did not differ in their fish species richness (mean ± SD: 37.8 ± 8.2; ANOVA: R^2^ = 0.111, F_2,7_ = 0.313, p = 0.745); however, we did detect significant differences in community species composition between natural and artificial reefs (PERMANOVA; Pseudo-F_1,7_ = 4.083, p = 0.001). Further analysis indicated that artificial reef communities were largely frequented by a subset of the overall community, most notably by Tomtate, Slippery Dick, Porkfish, Lionfish, Sharpnose Puffer, Purple Reef Fish, and French Grunt (SIMPER; mean Bray-Curtis dissimilarity between reef types was 55.3%; see Figures 1 and 2). The distance of the reef tract (whether artificial or natural) from shore was not found to significantly affect fish community composition (PERMANOVA; Pseudo-F_1,7_ = 1.263, p = 0.273), nor did it affect species richness or evenness (ANOVA; both p ≥ 0.473).

### 2.2 Stable isotope data

**Figure 3:**
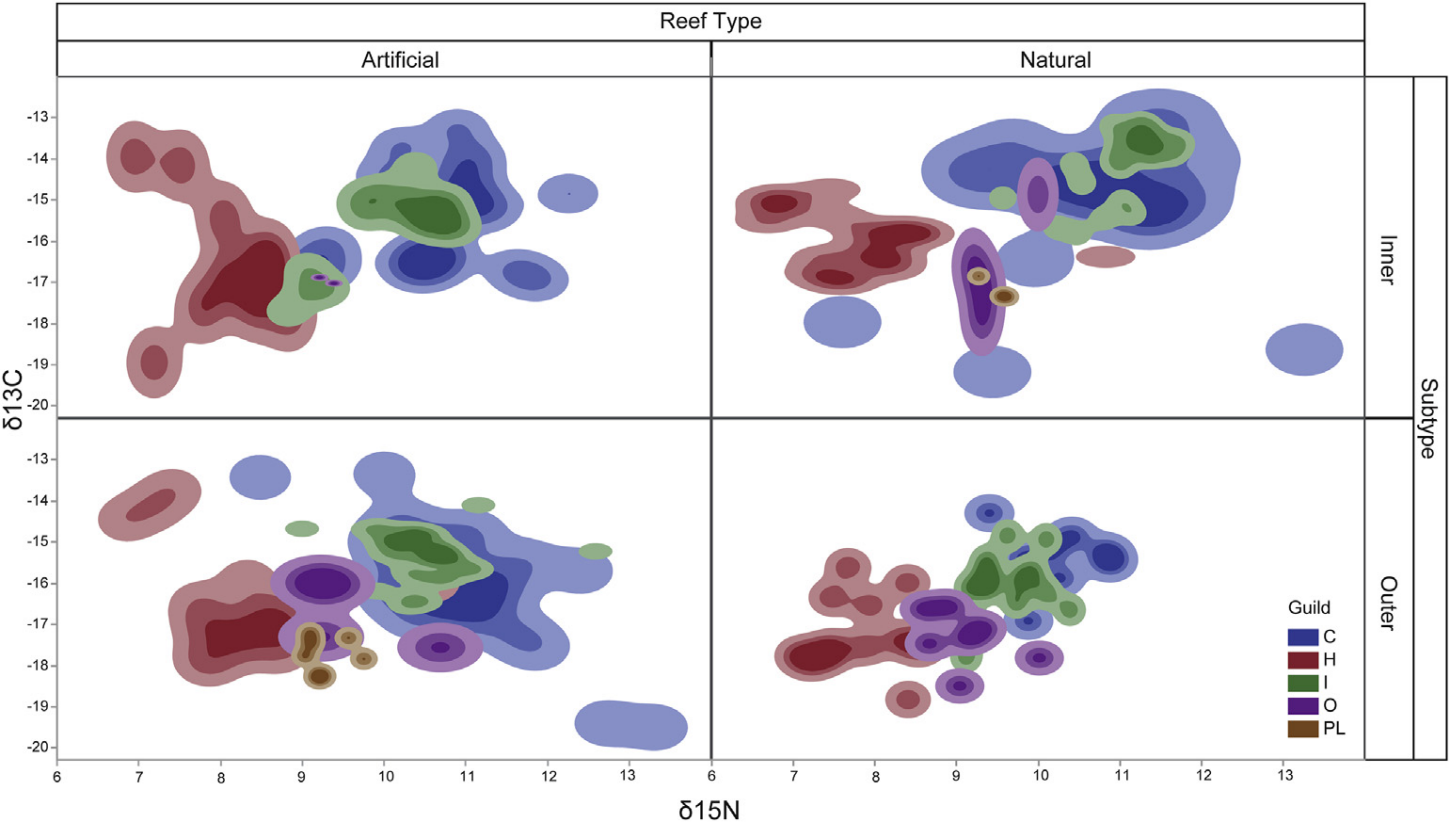
Density plot of δ^15^N and δ^13^C values recorded at inner and outer natural and artificial reefs. Values are color-coded by trophic guild. Trophic guild assignations for the various species are presented in Table 2. Although the overall stable isotope profiles varied among reef types (both inner and outer), our results indicate that these differences are being driven by that fact that these habitats are being used by different fish assemblages, and not by habitat-associated alterations in fish trophic behavior.

A total of 245 muscle tissue samples from 41 reef-associated fish species were analyzed for δ^15^N and δ^13^C (Table 1). Fish muscle stable isotope values for δ^15^N and δ^13^C ranged from 6.7 to 13.3‰ and -19.5 to -13.1 ‰, respectively. We tested the effect of trophic guild, family, standard length, reef type (natural vs. artificial), and reef subtype (inner vs. outer reef tract) on δ^15^N and δ^13^C values separately using ANOVA (Shapiro-Wilk testing confirmed normal distributions for both δ^15^N and δ^13^C data; δ^15^N: *P* < 0.01 and δ^13^C: *P* = 0.02; see Table 2). Family and trophic guild were the factors with the strongest effect size in both models. For δ^15^N, none of the other predictors were significant; however, for δ^13^C, we noted additional significant effects of reef subtype (i.e., the proximity of the reef to shore) and to a lesser extent, of reef type (Table 2). Fish standard length was not retained in either model. This was reflected in δ^15^N and δ^13^C density plots, which depicted variation among trophic guilds, reef type, and reef subtype that reflected differential use of artificial and natural reefs by various families and guilds of fishes (Figure 3).

**Table 1:** List of species sampled by common name, trophic position (TP; H: herbivore, O: omnivore, I: invertivore, C: carnivore), taxonomic family, number sampled (N), mean ($\bar{\text{x}}$) δ^15^N, δ^13^C, and standard length (in centimeters) ± standard deviation (SD). Also reported are the benthic algae (BA-TP) and phytoplankton (PP-TP) δ^15^N-based trophic position estimates, as well as the stomach content-based trophic position (SC-TP) estimates sourced from FishBase (Froese and Pauly 2016).

TP	Family	Species	N	$\bar{x}$ Length ±SD	$\bar{x\;}$ δ^15^N (‰) ±SD	$\bar{x\;}$ δ^13^C (‰) ±SD	BA-TP	PP-TP	SC-TP
H	Acanthuridae	*Acanthurus bahianus* Ocean Surgeon	16	20.9 ± 3.76	8.1 ± 0.43	-16.8 ± 0.46	2.7 ± 0.14	3.0 ± 0.14	2.0
H		*Acanthurus chirurgus* Doctorfish	6	25.1 ± 0.39	8.6 ± 0.27	-17 ± 0.70	2.9 ± 0.08	3.1 ± 0.08	2.0
H		*Acanthurus coeruleus* Blue Ttang	8	21.3 ± 3.49	8.0 ± 0.48	-17.6 ± 1.12	2.7 ± 0.15	2.9 ± 0.15	2.0
H	Scaridae	*Sparisoma aurofrenatum* Redband Parrotfish	12	15.0 ± 3.52	7.9 ± 0.57	-16.8 ± 0.97	2.6 ± 0.18	2.9 ± 0.18	2.0
H		*Sparisoma chrysopterum* Redtail Parrotfish	3	23.5 ± 1.52	8.0 ± 0.19	-17.2 ± 0.67	2.7 ± 0.06	2.9 ± 0.06	2.0
H		*Sparisoma viride* Stoplight Parrotfish	8	27.9 ± 8.03	7.3 ± 0.60	-15.4 ± 0.55	2.5 ± 0.19	2.7 ± 0.19	2.0
H	Pomacentridae	*Stegastes partitus* Bicolor Damselfish	5	6.1 ± 0.87	7.2 ± 0.30	-14.1 ± 0.27	2.4 ± 0.09	2.7 ± 0.09	2.0
O	Tetraodontidae	*Canthigaster rostrata* Sharpnose Puffer	2	6.9 ± 0.92	9.3 ± 0.12	-16.9 ± 0.1	3.1 ± 0.04	3.3 ± 0.04	3.3
O	Pomacanthidae	*Holacanthus ciliaris* Queen Angelfish	1	36.5	8.6	-16.6	2.9	3.1	3.0
O		*Holacanthus tricolor* Rock Beauty	3	16.1 ± 2.40	10.0 ± 0.69	-17.5 ± 0.37	3.3 ± 0.22	3.6 ± 0.22	3.0
O		*Pomacanthus paru* French Angelfish	5	30.6 ± 4.58	9.0 ± 0.25	-17.6 ± 0.71	3.0 ± 0.08	3.3 ± 0.08	3.1
O	Ostraciidae	*Rhinesomus triqueter* Smooth Trunkfish	1	11.0	10.0	-14.9	3.3	3.6	3.3
O	Pomacentridae	*Abudefduf saxatalis* Sergeant Major	5	16.0 ± 0.82	9.3 ± 0.08	-16.6 ± 0.6	3.1 ± 0.03	3.3 ± 0.03	3.8
I	Chaetodontidae	*Chaetodon capistratus* Foureye Butterflyfish	2	11.4 ± 0.99	10.2 ± 0.53	-15.3 ± 0.94	3.4 ± 0.17	3.6 ± 0.17	3.4
I		*Chaetodon sedentarius* Reef Butterflyfish	7	12.5 ± 0.89	10.2 ± 0.45	-16.3 ± 0.38	3.4 ± 0.14	3.6 ± 0.14	3.9
I	Diodontidae	*Diodon holocanthus* Balloonfish	2	17.3 ± 0.35	9.7 ± 0.27	-15.9 ± 0.39	3.2 ± 0.08	3.5 ± 0.08	3.3
I	Haemulidae	*Anisotremus virginicus* Porkfish	11	25.9 ± 2.56	10.7 ± 0.71	-15.2 ± 0.58	3.5 ± 0.22	3.8 ± 0.22	3.6
I		*Haemulon album* Margate	1	28.4	9.9	-14.8	3.3	3.5	3.3
I		*Haemulon aurolineatum* Tomtate	24	20.2 ± 1.58	10.5 ± 0.32	-15.2 ± 0.48	3.5 ± 0.10	3.7 ± 0.10	4.4
I		*Haemulon carbonarium* Caesar Grunt	2	25.0 ± 2.33	11.3 ± 0.21	-13.8 ± 0.03	3.7 ± 0.07	4.0 ± 0.07	3.7
I		*Haemulon flavolineatum* French Grunt	20	21.4 ± 3.42	11.2 ± 0.30	-13.7 ± 0.42	3.7 ± 0.09	4.0 ± 0.09	3.4
I	Balistidae	*Balistes capricsus* Gray Triggerfish	8	28.2 ± 1.15	9.2 ± 0.27	-17 ± 0.76	3.1 ± 0.09	3.3 ± 0.09	4.1
I	Labridae	*Bodianus rufus* Spanish Hogfish	3	28.3 ± 5.86	11 ± 0.18	-15.3 ± 0.21	3.6 ± 0.05	3.9 ± 0.05	3.7
I		*Halichoeres garnoti* Yellowhead Wrasse	3	12.3 ± 0.75	9.4 ± 0.11	-15.6 ± 0.60	3.1 ± 0.03	3.4 ± 0.03	3.7
I		*Lachnolaimus maximus* Hogfish	10	35.3 ± 4.46	9.9 ± 0.49	-15.2 ± 0.53	3.3 ± 0.15	3.5 ± 0.15	4.2
I	Sparidae	*Calamus proridens* Littlehead Porgy	1	31.3	10.2	-13.7	3.4	3.6	3.4
I	Tetraodontidae	*Sphoeroides spengleri* Bandtail Puffer	1	10.7	9.8	-15.4	3.3	3.5	3.5
C	Carangidae	*Carangoides bartholomaei* Yellow Jack	4	17.5 ± 1.01	10.7 ± 0.41	-14.7 ± 0.80	3.5 ± 0.13	3.8 ± 0.13	4.5
C		*Caranx crysos* Blue Runner	4	33.4 ± 3.20	11.0 ± 0.83	-16.5 ± 0.46	3.6 ± 0.26	3.9 ± 0.26	3.6
C		*Caranx ruber* Bar Jack	4	35.3 ± 0.32	9.2 ± 1.29	-17.5 ± 1.32	3.1 ± 0.40	3.3 ± 0.40	3.8
C		*Seriola rivoliana* Almaco Jack	8	38.6 ± 2.54	9.8 ± 0.61	-16.3 ± 0.81	3.3 ± 0.19	3.5 ± 0.19	4.5
C	Haemulidae	*Haemulon parra* Sailor's Choice	4	28.0 ± 2.85	10.9 ± 0.21	-14.3 ± 0.90	3.6 ± 0.06	3.9 ± 0.06	3.5
C		*Haemulon plumieri* White Grunt	9	23.2 ± 3.06	11.2 ± 0.34	-15.2 ± 1.23	3.7 ± 0.11	3.9 ± 0.11	3.8
C		*Haemulon sciuros* Bluestriped Grunt	11	20.3 ± 2.15	11.8 ± 1.13	-16.7 ± 1.92	3.9 ± 0.35	4.1 ± 0.35	3.4
C	Lutjanidae	*Lutjanus griseus* Gray Snapper	3	26.2 ± 1.33	11.1 ± 1.13	-14.2 ± 0.82	3.6 ± 0.35	3.9 ± 0.35	4.2
C		*Lutjanus synagris* Lane Snapper	1	25.0	11.1	-14.0	3.7	3.9	3.8
C		*Ocyurus chrysurus* Yellowtail Snapper	1	30.0	10.0	-16.5	3.3	3.5	4.0
C	Mullidae	*Pseudupeneus maculatus* Spotted Goatfish	5	17.8 ± 2.85	9.4 ± 0.27	-14.2 ± 0.31	3.1 ± 0.08	3.4 ± 0.08	3.7
C	Scorpaenidae	*Pterois* sp. Lionfish	7	20.1 ± 2.13	10.6 ± 0.30	-15.7 ± 0.83	3.5 ± 0.09	3.8 ± 0.09	4.4
C	Serranidae	*Cephalopholis cruentata* Graysby	11	25.1 ± 3.01	11.1 ± 0.53	-15.4 ± 0.52	3.6 ± 0.17	3.9 ± 0.17	4.3
C		*Hypoplectrus unicolor* Butter Hamlet	2	12.7 ± 0.71	10.5 ± 0.30	-14.4 ± 0.17	3.5 ± 0.09	3.7 ± 0.09	4.0

**Table 2:** Summary of ANOVA results for δ^15^N and δ^13^C values in fish muscle from South Florida artificial and natural reefs. Statistically significant factors are listed in bold, as well as LogWorth, a measure of the effect size associated with each factor.

Isotope	Factor	DF	F	*P*	LogWorth
δ^15^N	**Family**	**16**	**7.093**	**<0.001**	**12.561**
	**Trophic guild**	**4**	**6.420**	**<0.001**	**4.193**
	Reef type	1	0.003	0.935	0.021
	Distance from shore	1	1.886	0.171	0.767
	Body size	1	3.062	0.082	1.089
δ^13^C	**Family**	**16**	**7.944**	**<0.001**	**14.293**
	**Trophic guild**	**4**	**17.961**	**<0.001**	**12.151**
	**Reef type**	**1**	**11.051**	**<0.001**	**2.988**
	**Distance from shore**	**1**	**14.139**	**<0.001**	**3.688**
	Body size	1	0.466	0.495	0.305

## 3 Discussion

Our primary conclusion, based on comparative analysis of the catch and survey data, is that fish community composition differed significantly among reef types, and that this in turn shaped the overall stable isotope profiles at each location (Figure 3). Furthermore, δ^15^N was significantly influenced by its trophic guild (i.e., feeding strategy) but not by the artificial or natural state of the reef where it was collected – in other words, the stable isotope profiles of each fish taxon reflected their feeding ecology rather than reef type. We saw no evidence that fishes adjusted their feeding preference depending on the reef type from which they were sampled. Lastly, δ^13^C was significantly influenced by both trophic guild and by the distance from shore at which the individual was sampled, suggesting that there are differences between these communities (first reef tract, second reef tract, and artificial reefs) in their basal carbon sources.

### 3.1 Natural and artificial reefs differ in fish species composition

Species composition was significantly influenced by reef type, suggesting that the fish communities of the artificial reef sites and natural reef sites differed in their species composition. Previous studies in the region have shown that the fish assemblages of the first and second reef tracts are different (Ault et al., 2001; Moyer et al., 2003; Ferro et al., 2005). Additionally, fish assemblages of artificial reefs are known to vary with depth, structural complexity, refuge size, and relief height (Hixon and Beets, 1989; Sherman et al., 2001, 2002; Walker et al., 2002; Arena et al., 2007).

### 3.2 δ^15^N and δ^13^C varied among fish families and trophic guilds

The δ^15^N and δ^13^C values of muscle tissue were primarily influenced by *family* and *trophic guild.* The results are also consistent with prior studies which found dietary habits directly affect δ^15^N and δ^13^C, and that higher trophic level feeding behavior results in enrichment of δ^15^N and δ^13^C (e.g., Post, 2002; Mill et al., 2007; Cresson et al., 2014). Members of the 17 families sampled for this study shared similar morphological and ecological traits which were reflected in the similarity of their feeding behaviors (Greenwood et al., 2010) and isotopic signatures (Supplemental Materials).

### 3.3 Herbivores

The calculated δ^15^N-based trophic position estimates were mostly similar to the stomach content-based trophic position estimates, except for those species in the trophic guild *herbivore*. We found the members of this guild to be the most depleted in δ^15^N, which is consistent with other studies that show that primary consumption tends to result in more depleted δ^15^N relative to higher trophic level feeders (DeNiro and Epstein, 1981; Greenwood et al., 2010; Goldman et al., 2016). The average δ^13^C for this guild was -16.5‰, which is consistent with diets of marine benthic marine algae (France, 1995a); this agreed with δ^15^N-based trophic position estimates calculated using marine benthic algae that more closely match the stomach content-based trophic position estimates, making it the most likely food source. The δ^15^N-based trophic position estimates were slightly higher than the stomach content-based trophic position estimates which is consistent with the findings of Cresson et al. (2014). The slight enrichment may be a result of detritus consumption or simply that the fractionation rate may be different between herbivores and higher trophic level feeders due to slight differences in their respective enzymatic and digestive systems (Mill et al., 2007). Of the other three primary producers considered, the δ^13^C range of phytoplankton (-22 to -17‰) most closely resembles the δ^13^C of benthic marine algae, which makes it difficult to distinguish the two primary producers (France, 1995a; Kieckbusch et al., 2004). It is unlikely, however, that phytoplankton is the dominant source of carbon for individuals within the trophic guild *herbivore* as these fishes predominantly graze on benthic marine algae. Mangroves were initially considered as a possible basal carbon source, but the δ^13^C range (-30 to -24‰) was too depleted, so mangroves were excluded. Seagrasses were also considered as a possible food source, but the exhibited δ^13^C range (-13 to -7‰) was much more enriched than the herbivores collected in this study, with the exception of the Bicolor Damselfish. Emery (1973) noted the diet of damselfish in the Florida Keys to be planktivorous/omnivorous, potentially accounting for this enrichment (Supplemental Material, Table 1). Seagrass beds of Broward County, Florida are limited to the Inter-Coastal Waterway (ICW) (Walker, 2012). Gabriel et al. (2015) found that seagrass beds within the ICW had a mean δ^15^N of 5.6‰, which was too enriched to be the basal carbon source for the Bicolor Damselfish of this study. France and Holmquist (1997) found that in areas with decreased water movement, benthic marine algae can be enriched in δ^13^C by as much as 9‰. It may be that the complex structure of the artificial reef piles where the Bicolor Damselfish were sampled reduced water movement enough to cause the algal food source to become more enriched in δ^13^C.

### 3.4 Omnivores

Species of the trophic guild *omnivore* were slightly more enriched in δ^15^N (9.3‰ ± 0.5) and more depleted in δ^13^C (-17.0‰ ± 0.85) when compared to those in the trophic guild *herbivore* (Figure 1 A-B). The trophic guild *planktivore* had δ^15^N and δ^13^C that were similar to those of the trophic guild *omnivore*, suggesting that they utilize similar food sources. The δ^13^C (-17.6‰ ± 0.46) of trophic guild *planktivore* suggests that phytoplankton is the source of primary production in the diet of these species. Additionally, the δ^15^N-based trophic position estimates using phytoplankton as the food web base more closely matches the stomach content-based trophic position when compared to the other primary producers. Phytoplankton tends to exhibit δ^15^N that is less enriched when compared to marine benthic algae (Cresson et al., 2014), which would explain why the mean δ^15^N of the trophic guild *planktivore* are not as enriched as the trophic guild *invertivore*.

### 3.5 Invertivores

The trophic guild *invertivore* was more enriched in δ^15^N (10.5‰ ± 0.74) relative to the other trophic guilds in this study, with the exception of the trophic guild *carnivore*, which is consistent with higher trophic level feeding habits relative to the other trophic guilds of this study. Species within the trophic guild *invertivore* are known to feed primarily on marine invertebrate fauna, and Behringer and Butler (2006) found that marine benthic algae is an important food resource for benthic invertebrates on the reef systems of southeast Florida. For this trophic guild, δ^15^N-based trophic position estimates using marine benthic algae as the food web base were closest to the stomach content-based trophic positions.

### 3.6 Carnivores

The trophic guild *carnivore* consists of reef-associated fish species that exhibit a diet of both marine invertebrates and teleost fishes. Piscivory (exclusive consumption of fishes) is associated with higher trophic level feeding, and it was expected for this reason that individuals within this guild would exhibit the highest levels of enrichment in δ^15^N (Cresson et al., 2014). While this trophic guild does exhibit the highest mean enrichment in δ^15^N (10.7‰ ± 1.01), it is only slightly more enriched compared to the mean δ^15^N of the trophic guild *invertivore* (10.5‰ ± 0.74). Additionally, the mean δ^13^C of the trophic guild *carnivore* (15.5‰ ± 1.4) is similar to the mean δ^13^C of the trophic guild *invertivore* (15.1‰ ± 1.07), suggesting that the individuals of these two trophic guilds share similar feeding habits. The mean δ^13^C of species within this guild suggest that marine benthic algae are the major carbon source for their diets (Figure 1 C-D).

### 3.7 Offshore vs inshore reefs

The GLM found that the δ^13^C of samples were significantly influenced by reef type (artificial versus natural) and distance from shore (inner versus outer). The mean δ^13^C for these locations (natural first reef: -15.1‰, natural second reef: -16.5‰, inner artificial: -15.8‰, outer artificial: -16.0‰) increased slightly with seaward movement. This trend is consistent with prior studies that reported increasingly depleted δ^13^C values of sampled fauna with seaward movement and depth (France, 1995a, 1995b; Bouillon et al., 2008; Wyatt et al., 2012) (Figure 2). Additionally, the trophic guild *herbivores* made up a larger percentage of the catch composition of the second reef sites compared to the other location types (Figure 2), which would also lower the mean δ^13^C of the second reef sites. Alternatively, it may be that transitory movement between the first and second reef is the root cause for samples from the artificial reefs having intermediate δ^13^C. With the exception of Pomacentrids, which display territorial behavior, the fishes of this study are active foragers and grazers, moving over the reef in search of food (Valdés-Muñoz and Mochek, 2001). As an example, this study found Bluestriped Grunts on the first reef and artificial reef sites that had δ^15^N and δ^13^C values suggesting they were feeding in inshore mangrove forests. As reported by Lindberg et al. (2006), artificial reefs can be utilized solely as shelter, and it may be that the fishes of this study are utilizing the artificial reef piles as shelter as they transition between the first and second reef. If these fishes were feeding on both the first and second reef, isotopic mixing would explain why these fishes displayed intermediate δ^13^C.

### 3.8 Other factors

Unexpectedly, δ^15^N and δ^13^C values were not significantly linked to body size. In prior studies, body size has been shown to influence an individual's diet through secondary factors such as gape dimensions and swimming speed (Greenwood et al., 2010). Additionally, diet shifts correlated to body size have been observed in numerous marine fish species (Jennings et al., 2001). However, Al-Habsi et al. (2008) reported a similar lack of relationship between body size and δ^15^N and δ^13^C in a demersal fish community in the Arabian Sea. For this study, it is likely that body size was not a significant factor influencing the δ^15^N and δ^13^C of muscle tissue samples because of the similar size ranges among individuals within the trophic guilds.

### 3.9 Conclusion

Artificial reefs are intended to supplement natural benthic habitat for the purpose of enhancing biological production of marine life. The goal of this study was to compare the habitat use and feeding behaviors of reef-associated fishes at both artificial limestone boulder habitats and natural reef habitats through the use of community survey stable isotope ecology. Although the community structure of these sampled reef-associated fishes differed between the artificial and natural reefs, this did not impact the respective trophic relationships. Species that generally follow low trophic level feeding strategies (i.e., herbivory) had the lowest δ^15^N, with δ^15^N increasing with higher trophic level feeding. Overall, the trophic relationships of fishes from the artificial reefs were similar to the natural reef sites, which suggests that both are offering similar food resources.

The stable isotopic trends observed in the reef fish communities of the artificial reef and the adjacent first and second reef tract suggest that the artificial reefs are acting as a corridor between the first and second reef, with opportunistic feeding occurring at the artificial reef sites. It is well established that connectivity between reef habitats is important for overall reef health and in this capacity, the artificial reefs studied here seem to increase connectivity and simultaneously provide opportunistic foraging habitat.

## 4 Limitations of the study

Fish community composition data collection was limited to two surveys completed within one year. During these surveys, two members of a three-person dive team identified 83 different fish species across 17 families. Spearguns were used to acquire fish samples for stable isotope analysis, which inherently limited the size range of the fishes that could be sampled.

## Declarations

### Author contribution statement

Christopher Blanar: Analyzed and interpreted the data; Contributed reagents, materials, analysis tools or data; Wrote the paper.

Joseph Hornbeck & David William Kerstetter: Performed the experiments; Analyzed and interpreted the data; Contributed reagents, materials, analysis tools or data; Wrote the paper.

Amy Hirons: Conceived and designed the experiments; Analyzed and interpreted the data; Wrote the paper.

### Funding statement

This work was supported by 10.13039/100006596Florida Fish and Wildlife Conservation Commission (FWC-11229).

### Data availability statement

Data will be made available on request.

### Declaration of interests statement

The authors declare no conflict of interest.

### Additional information

No additional information is available for this paper.

## Supplemental Information: Transparent Methods

### *Study sites*

The artificial reef, composed of 12, *ca.*1 m^3^ concrete boulders, was constructed in October 2009; sampling took place between July 23 and August 29, 2014. Therefore, infaunal and benthic habitats had developed over nearly 5 years. Fish were sampled from 4 artificial reef sites at an average depth of 13 m (m), located equidistant between the first and second natural reef tracts. Studies have shown that the physical characteristics and fish assemblages of the first and second reef tract are different (Ault et al., 2001; Moyer et al., 2003; Ferro et al., 2005), which may impact the feeding dynamics of those sites. As the δ^13^C values of sampled fauna often become more depleted in the seaward direction (France, 1995a, 1995b; Wyatt et al., 2012), the artificial reef sites were compared to both the first and second reef tracts. Two sites for the adjacent first reef tract averaged a depth of 6 m while 2 sites from the adjacent second reef tract averaged a depth of 16 m. To avoid potential differences in trophic relationships between the wet and dry seasons of southeast Florida (Misra and DiNapoli, 2013), sampling was only conducted during the wet season (June through September).

### *Fish collection and test for sampling bias*

The Institutional Animal Care and Use Committee (IACUC) at Nova Southeastern University reviewed and issued IACUC Control # 040-468-12-0912 for this study. Specimen collections were conducted under Florida Fish and Wildlife Conservation Commission (FWC) permit number SAL-13-1537.

Reef fish collection was conducted on SCUBA equipment using a spear gun for approximately 20 min to standardize sampling effort among the sites. In addition to fish collection, we also conducted a visual census of the entire fish assemblage at each sample site; the purpose of this census was to assess the extent to which our collection method might be biased, for example by missing bottom-associated herbivores and invertivores. Using the survey methods of Bannerot and Bohnsack (1986), divers observed faunal species diversity and abundance for 15 min at each sample site. Survey cylinder dimensions were standardized at a diameter of approximately 20 m and a relief height of 5 m. Fish species diversity and abundance were recorded using the Reef Environmental Education Foundation (REEF) Fish Survey Project's methodology; counts of observed species were assigned to 1 of 4 log_10_ abundance categories: single (1), few (2–10), many (11–100), and abundant (>100) (Pattengill-Semmens and Semmens, 2003). Density scores were calculated for each species by site using abundance categories and the Eq. (1):

(1) D = [(nSx1) + (nFx2) + (nMx3) + (nAx4)] / (nS + nF + nM + nA)

where D is the density score and nS, nF, nM, and nA are the number of times an abundance category was given (Pattengill-Semmens and Semmens, 2003). We calculated Bray-Curtis similarities for all pairs of sample sites for our sample collection and visual assessment data, and used a mantel test (the RELATE procedure in PRIMER-e) to compare their fish communities. The resulting rho value of 0.658 indicates that the spearfishing method used for sample collection missed some species that were visually detected, most likely smaller-sized, bottom-associated fishes. While acknowledging this sampling bias, statistical analysis indicated that the communities of fishes collected for analysis using spearfishing was broadly and significantly representative of the total fish communities at each site (RELATE; ρ = 0.658, p = 0.004).

### *Fish processing*

Each fish was measured (standard length to the nearest centimeter, cm) and weighed (total mass to the nearest gram, g). Approximately 30 g of white muscle tissue (Pinnegar and Polunin, 1999) was taken from the anterior dorsal region and processed for stable isotope analysis. We opted to analyze muscle instead of other tissues (e.g., blood) because muscle isotopes tend to reflect the whole organism and are thus more commonly reported in the literature, making it easier to compare and contextualize our results; furthermore, muscle isotopes reflect longer term feeding patterns than tissues such as blood, which have higher turnover rates. Excised muscle tissue was dried at 60 °C for a minimum of 72 h, homogenized, weighed, and pelletized. Stable isotope analysis was conducted using a Finnigan Delta Plus continuous flow isotope ratio mass spectrometer (CF-IRMS) at the Smithsonian OUSS/MCI Stable Isotope Mass Spectrometry Laboratory (Suitland, MD). All samples were linearly corrected with a two-point correction to acetanilide and urea standards calibrated to a Vienne-Pee Dee Belemnite (V-PDB) standard; V-PDB was the standard used for ^13^C/^12^C, and atmospheric air for ^15^N/^14^N. Reproducibility was 0.2‰. The ratio of the heavy to light isotopes, expressed as δ, for each tissue sample was calculated and expressed using the Eq. (2):

(2) δ (‰) = [(R_sample_ ∗ R_standard_) - 1] ∗ 1000

Trophic position estimates were calculated for each sample using its δ^15^N and the Eq. (3) (Post 2002):

(3) Trophic position = λ + (δ^15^N_Consumer_ - δ^15^N_Base_) /Δ_n_

where λ is the trophic level of the organism used as the δ^15^N_Base_ and Δ_n_ is the rate of δ^15^N enrichment per trophic interaction. The rate of enrichment (Δ_n_) was set at 3.2‰ per Sweeting et al. (2007). The dominant marine primary producers in southeastern Florida are mangroves, benthic algae, phytoplankton, and seagrasses (Kieckbusch et al., 2004), and these producers were considered as potential δ^15^N_Base_. The δ^13^C of sampled fishes were used to indicate the initial source of carbon (i.e., the food web base) (DeNiro and Epstein, 1978; Tieszen et al., 1983; Peterson and Fry, 1987). The δ^15^N of seagrasses (Gabriel et al., 2015) and red mangrove *Rhizophora mangle* (Parks, 2013) were sourced from studies conducted in Broward County, Florida (5.6‰ and 2.7‰, respectively. Benthic macroalgae δ^15^N (2.6‰) was sourced from a study performed in southeast Florida by Behringer and Butler (2006) and phytoplankton δ^15^N (1.8‰) from Rau et al. (1990). The δ^15^N trophic position estimates were compared to stomach content-based trophic position estimates for each species sourced from FishBase (model details in Froese et al., 1992; estimate sources from Froese and Pauly 2016).

### *Trophic Guild Assignment*

Species of this study were assigned to trophic guilds (Table S1) based on dietary habits, which were sourced via literature review. The trophic guild *herbivore* consisted of those species that typically consume marine flora, the trophic guild *planktivore* on plankton, and the trophic guild *omnivore* displayed primary and secondary consumption. More specifically, the trophic guild *invertivore* fed on benthic invertebrates and the trophic guild *carnivore* consisted of those species that fed on marine invertebrates as well as fishes. These guilds are also noted for each study species in Table 1.

### *Data Analysis*

PRIMER (version 7.0.9; PRIMER-E, Ltd.; Ivybridge, UK) was used to calculate among-site Bray-Curtis fish community similarity indices for both survey and collection data, which were used to establish triangular matrices of fish community similarity. Community composition data (with species abundances summed by site) were then examined with a Permutational Multivariate Analysis of Variance (PERMANOVA) to assess the extent to which the species composition of sites was affected by reef type (natural versus artificial) and distance from shore (inner versus outer).

Analysis of Variance (ANOVA) in JMP (version 10.0; SAS, Cary NC, USA) was used to examine differences in δ^15^N and δ^13^C values of individual muscle tissue samples as a function of *family* (17 levels, individual fish family taxa), *trophic guild* (4 levels: *herbivore*, *omnivore*, *invertivore*, and *carnivore*; samples belonging to *planktivore* were not used in this analysis because they were not present at all three locations), *reef type* (2 levels: artificial and natural), *distance from shore* (2 levels: inner and outer), and *standard length*. The factors *family*, *trophic guild*, and *size* were used to test whether these factors influence δ^15^N and δ^13^C. The factors *habitat type* and *distance from shore* were used to compare the trophic dynamics of the artificial reefs against the first and second natural reef tracts. Statistical significance was evaluated at α = 0.05.

## Appendix A Supplementary data

Heliyon reef SIA revisions_Table S1 -- June 2021 Heliyon reef SIA revisions_Table S1 -- June 2021
