# Supplementary material for: Stable isotopes and community surveys reveal differential use of artificial and natural reefs by South Florida fishes
Source: Heliyon. 2021 Jun 26;7(7):e07413. doi: 10.1016/j.heliyon.2021.e07413 (PMC8273218; doi:10.1016/j.heliyon.2021.e07413)
Supplement: Heliyon reef SIA revisions_Table S1 -- June 2021 [file mmc1.docx]

Table S1. Trophic Guild (TG) categorization used in this study based on the general feeding strategy of each reef-associated fish species encountered at artificial or natural reef sites in southeast Florida. Prey items were sourced from primary literature. Fish species are listed by scientific name and trophic code (TC).

| **TG** | **TC** | **Species** | **Prey items** | **Reference(s)** |
| --- | --- | --- | --- | --- |
| Herbivore | H_1_ | *Acanthurus bahianus* (Castelnau, 1855) | algae, phanerogams | Randall 1967 |
|  | H_2_ | *Acanthurus* *chirurgus* (Bloch, 1787) | algae | Randall 1967 |
|  | H_3_ | *Acanthurus coeruleus* (Bloch & Schneider, 1801) | algae | Randall 1967 |
|  | H_4_ | *Sparisoma aurofrenatum* (Valenciennes, 1840) | sponge, algae, phanerogams | Randall 1967,  Dunlap and Pawlik 1998 |
|  | H_5_ | *Sparisoma chrysopterum* (Bloch & Schneider, 1801) | sponge, algae, phanerogams | Randall 1967,  Dunlap and Pawlik 1998 |
|  | H_6_ | *Sparisoma* *viride* (Bonnaterre, 1788) | algae, phanerogams | Randall 1967 |
|  | H_7_ | *Stegastes partitus* (Poey, 1868) | algae, copepods, shrimp | Randall 1967, Emery 1973 |
| Omnivore | O_1_ | *Canthigaster rostrata* (Bloch, 1786) | phenerogams, sponge | Randall 1967 |
|  | O_2_ | *Holacanthus ciliaris* (Linnaeus, 1758) | algae, sponge, tunicates, hydrozoans | Randall 1967 |
|  | O_3_ | *Holacanthus* *tricolor* (Bloch, 1787) | algae, zoantharians, sponge | Randall 1967 |
|  | O_4_ | *Pomacanthus* *paru* (Bloch, 1787) | algae, sponge, tunicates, zoantharians, gorgonians | Randall 1967 |
|  | O_5_ | *Rhinesomus* *triqueter* (Linnaeus, 1758) | annelids, sipunculids, crabs, shrimps, tunicates, sponge | Randall 1967 |
|  | O_6_ | *Abudefduf* *saxatilis* (Linnaeus, 1758) | anthozoans, copepods, algae, tunicates | Randall 1967 |
| Invertivore | I_1_ | *Chaetodon* *capistratus* (Linnaeus, 1758) | zooantharians, annelids, gorgonians, tunicates | Randall 1967, Lasker 1985 |
|  | I_2_ | *Chaetodon* *sedentarius* (Poey, 1868) | annelids, shrimps, amphipods, | Randall 1967 |
|  | I_3_ | *Diodon holocanthus* (Linnaeus, 1758) | gastropods, pelecipods, sea urchins, Crabs | Randall 1967 |
|  | I_4_ | *Anisotremus* *virginicus* (Linnaeus, 1758) | sea urchin, crabs, shrimps, annelids, pelecipods | Randall 1967 |
|  | I_5_ | *Haemulon album* (Cuvier, 1830) | crabs, shrimps, sea urchins, annelids | Cummings et al. 1966, Randall 1967 |
|  | I_6_ | *Haemulon* *aurolineatum*  (Cuvier, 1830) | shrimps, annelids, crabs, amphipods, pelecipods | Randall 1967 |
|  | I_7_ | *Haemulon* *carbonarium* (Poey, 1868) | crabs, gastropods, sea urchin, annelids | Randall 1967 |
|  | I_8_ | *Haemulon* *flavolineatum* (Desmarest, 1823) | annelids, crabs, holothurians, shrimps, pelecipods | Randall 1967 |
|  | I_9_ | *Balistes* *capriscus* (Gmelin, 1789) | mollusks, crustacea | Goldman et al. 2016 |
|  | I_10_ | *Bodianus* *rufus* (Linnaeus, 1758) | crabs, ophiuroids, sea urchins, gastropods | Randall 1967 |
|  | I_11_ | *Halichoeres* *garnoti* (Valenciennes, 1839) | crabs, ophiuroids, gastropods, | Randall 1967 |
|  | I_12_ | *Lachnolaimus* *maximus* (Walbaum, 1792) | gastropods, crabs, ophiuroids | Randall 1967 |
|  | I_13_ | *Calamus* *proridens* (Jordan & Gilbert, 1884) | crustaceans | Druzhinin 1976 |
|  | I_14_ | *Sphoeroides* *spengleri* (Bloch, 1785) | crabs, mollusks, annelids, echinoids | Randall 1967 |
| Carnivore | C_1_ | *Carangoides* *bartholomaei* (Cuvier, 1833) | fishes, cephalopods, shrimps | Randall 1967, Sierra et al. 1986 |
|  | C_2_ | *Caranx* *crysos* (Mitchill, 1815) | fishes, cephalopods, crabs, stematopods | Randall 1967 |
|  | C_3_ | *Caranx* *ruber* (Bloch, 1793) | fishes | Randall 1967, Sierra and Popova 1982 |
|  | C_4_ | *Seriola* *rivoliana* (Valenciennes, 1833) | fishes, cephalopods, | Manooch and Haimovici 1983 |
|  | C_5_ | *Haemulon* *parra* (Desmarest, 1823) | shrimps, crabs, amphipods, gastropods, annelids, fishes | Randall 1967 |
|  | C_6_ | *Haemulon* *plumieri* (Lacepède, 1801) | crabs, annelids, sea urchins, gastropods, fishes | Bowman et al. 2000 |
|  | C_7_ | *Haemulon* *sciurus* (Shaw, 1803) | crabs, pelecipods, shrimps, sea urchins, fishes | Randall 1967 |
|  | C_8_ | *Lutjanus* *griseus* (Linnaeus, 1758) | fishes, crabs, shrimps | Starck 1971, Claro 1983a |
|  | C_9_ | *Lutjanus* *synagris* (Linnaeus, 1758) | fishes, crabs, shrimps | Randall 1967, Claro 1981 |
|  | C_10_ | *Ocyurus* *chrysurus* (Bloch, 1791) | fishes, crabs, shrimps | Claro 1983b |
|  | C_11_ | *Pseudupeneus* *maculatus* (Bloch, 1793) | crabs, shrimps, annelids, mollusks, fishes | Randall 1967 |
|  | C_12_ | *Pterois* *volitans* (Linnaeus, 1758) | fishes, shrimps, crabs | Sano 1984, Morris 2009 |
|  | C_13_ | *Cephalopholis* *cruentata* (Lacepède, 1802) | fishes, stomatopods, crabs, gastropods | Randall 1967 |
|  | C_1_ | *Hypoplectrus* *unicolor* (Walbaum, 1792) | crustaceans, fishes | Cervigón et al. 1994 |
